# EPO Receptor Blockade in Dendritic Cells Enhances Antitumor CD8^+^ T Cell Immunity

**DOI:** 10.34133/cancomm.0027

**Published:** 2026-05-14

**Authors:** Yunfei Zhu, Xuetao Cao

**Affiliations:** ^1^ Department of Immunology, Institute of Basic Medical Sciences, Peking Union Medical College, Chinese Academy of Medical Sciences, Beijing 100005, P. R. China.; ^2^ Institute of Immunology, College of Life Sciences, Nankai University, Tianjin 300071, P. R. China.

Dendritic cells (DCs) bridge innate and adaptive immunity by initiating and directing antigen-specific immune responses. Among DC subsets, type 1 conventional DCs (cDC1s) are particularly vital for immunity against tumors and viral infections due to their unique ability to cross-present antigens to CD8^+^ T cells. cDC1s engulf antigens through efferocytosis of apoptotic cells or uptake of immunogenic necrotic material [1]. This process triggers their maturation and migration to lymph nodes, where they present antigens to T cell subsets and ultimately guide the immune response toward either immunostimulatory or tolerogenic outcomes and set its intensity [2]. Specifically, cDC1s can activate both CD8^+^ and CD4^+^ T cells to drive immunity, representing an immunostimulatory maturation program [3]. However, they can also prime antigen-specific forkhead box P3-positive regulatory T cells (Tregs) to establish immune tolerance, reflecting a distinct tolerogenic maturation program [3]. This functional divergence has major disease-specific consequences, as immune tolerance provides benefits in transplantation and autoimmunity while proving detrimental in cancer by suppressing antitumor immunity [4]. Despite extensive work defining the definite functions of cDC1s in various microenvironments, the upstream mechanisms that determine whether cDC1s adopt an immunostimulatory or tolerogenic maturation program still remain unclear.

In a recent study, Zhang et al. [5] addressed this fundamental question by identifying the erythropoietin receptor (EPOR) as a central regulator of cDC1 tolerogenic programming. Firstly, to investigate how cDC1s contribute to immune tolerance, the authors employed a tolerized model system combining total lymphoid irradiation (TLI), antithymocyte serum (ATS), and allogeneic donor bone marrow infusion. cDC1s were found to be essential in this setting. Transcriptomic analysis revealed that cDC1s from tolerized mice up-regulated *Epor* along with enrichment of mechanistic target of rapamycin (mTOR)signaling, as well as genes associated with efferocytosis and lipid metabolism. Conditional knockout of *Epor* in cDC1s (*Epor^ΔXcr1^*) abolished mixed chimerism and led to rapid allograft rejection, demonstrating that EPOR on cDC1s is necessary for tolerance induction. Mechanistically, the allogeneic bone marrow infusion experiment and in vitro coculture assay showed that EPOR is required for de novo differentiation of naïve T cells into antigen-specific Tregs as well as for expansion of preexisting Treg populations. These findings identify cDC1-intrinsic EPOR signaling as a key pathway driving antigen-specific immune tolerance.

Subsequently, to further investigate the mechanisms of cDC1-mediated immune tolerance via EPOR signaling, single-cell transcriptomic analyses were conducted to reveal that EPOR signaling is required to control the functional maturation of cDC1s. Under tolerogenic conditions, cDC1s progress through distinct early and C-C chemokine receptor type 7-positive (CCR7^+^) late-mature states with reduced cross-presentation capacity, representing enhanced tolerogenic maturation. Meanwhile, EPOR-deficient cDC1s displayed attenuated efferocytosis-triggered tolerogenic maturation and instead increased expression of immunostimulatory costimulatory molecules upon mature signals, enhancing priming of CD8^+^ and CD4^+^ effector T cells. Notably, beyond marking a stable population, EPOR signaling actively programmed a unique tolerogenic transcriptome, with up-regulation of genes, such as integrin subunit beta 8 (*Itgb8*) and aldehyde dehydrogenase 1 family member A2 (*Aldh1a2*). Functional studies further showed that cDC1-specific deletion of *Itgb8*, but not *Aldh1a2*, severely impaired antigen-specific Treg induction and transplant tolerance, establishing *Itgb8* as a key downstream effector. Thus, EPOR signaling programs a tolerogenic functional state in cDC1s while simultaneously restraining their intrinsic immunostimulatory programming.

Beyond the spleen, EPOR expression is detected on a subset of migratory cDC1s in peripheral lymph nodes (PLNs). These EPOR^+^ migratory cDC1s exhibit enhanced capacity to induce antigen-specific Tregs in vitro, and their expression of EPOR is required for optimal Treg induction in PLNs; this function is further potentiated by erythropoietin (EPO) in an EPOR-dependent manner. Notably, this expression pattern was conserved across all PLNs examined, indicating that cDC1s acquire the EPOR signal before entering the lymph nodes. These findings collectively establish the EPO–EPOR axis as a key regulator and define a migratory tolerogenic cDC1 population.

Finally, the authors applied these insights to cancer immunotherapy via EPOR blockade in cDC1s. EPOR was expressed on tumor-infiltrating CCR7^+^ cDC1s and tumor antigen-carrying migratory cDC1s in the tumor-draining lymph nodes, which can potentially promote tumor-specific tolerance. Loss of EPOR in cDC1s enhances antitumor immunity, leading to an approximately 2- to 3-fold reduction in MC38-OVA and B16F10-OVA tumor growth relative to controls. This is accompanied by increased priming of tumor-specific CD8^+^ T cells, expansion of effector and T cell factor 1-positive (TCF1^+^) precursor-exhausted T cell populations, and reduced accumulation of Tregs. Notably, EPOR deficiency synergizes with programmed cell death protein 1 blockade, identifying EPOR as a cDC1-intrinsic immune checkpoint that sustains tumor immune tolerance.

In summary, EPOR acts as a key regulator in cDC1s, dictating whether they adopt tolerogenic or immunostimulatory functional programming (Fig. 1). Specifically, in tolerogenic contexts marked by apoptotic cells and high EPO levels, EPOR expression drives CCR7^+^ late-stage maturation and integrin β8-dependent induction of antigen-specific Tregs, while restraining effector T cell priming. Conversely, loss of EPOR signaling unleashes the immunostimulatory potential of cDC1s. Previous work from the same group demonstrated that tumor-derived EPO promotes an immunosuppressive tumor microenvironment through EPOR signaling in macrophages [6]. These findings further establish the EPO–EPOR axis as an essential signal in cDC1, which coordinates immune tolerance in transplantation and tumor immune evasion, thereby highlighting EPOR as a promising target for modulating antigen-specific immune responses.

**Fig. 1.**
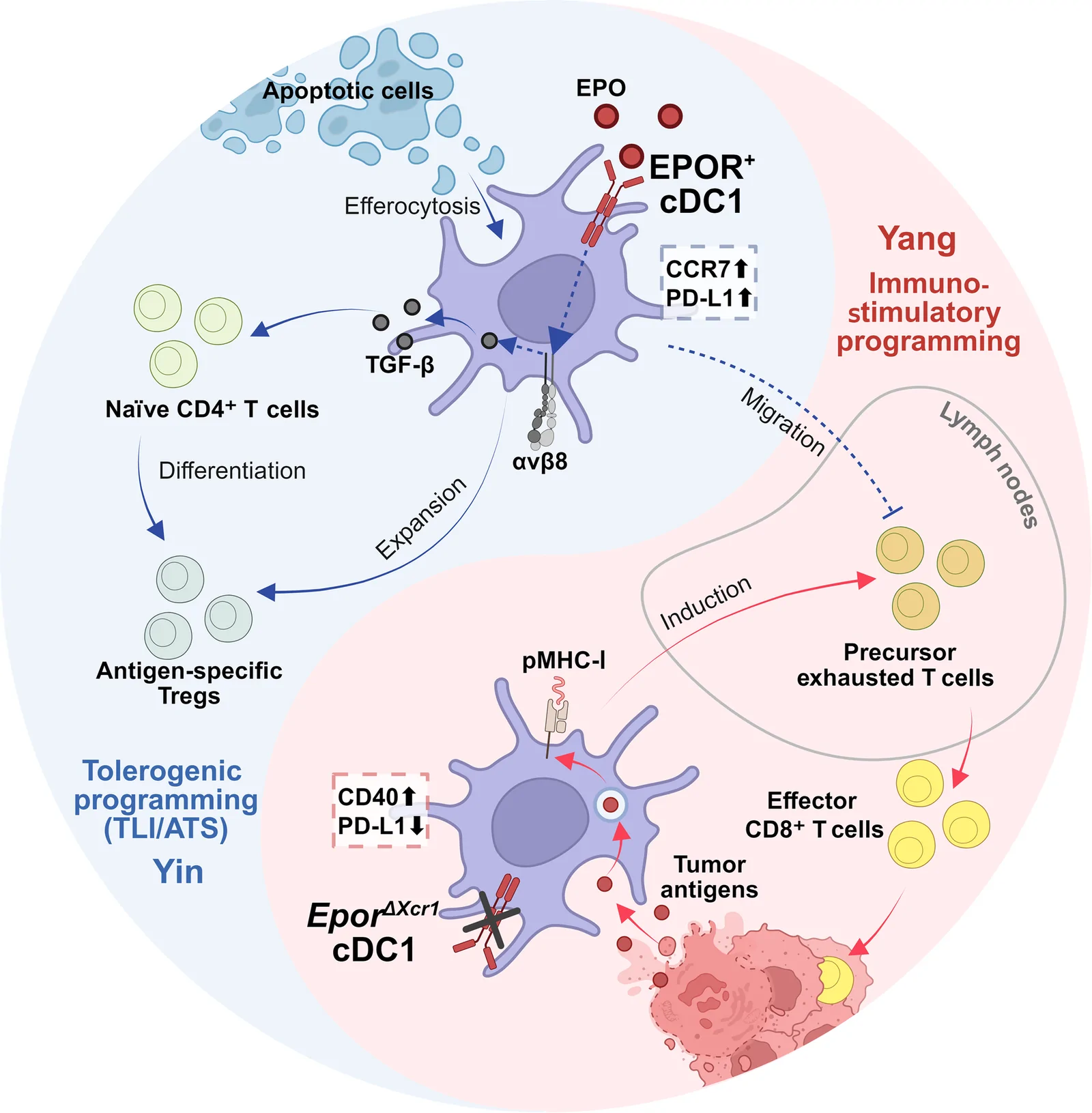
EPOR acts as a key regulator of tolerogenic and immunostimulatory programming in cDC1s. EPOR signaling in cDC1s determines their functional maturation and downstream immune outcomes. Specifically, in the TLI/ATS-based model, upon efferocytosis of apoptotic cells, EPOR^+^ cDC1s undergo tolerogenic maturation, marked by increased expression of CCR7, PD-L1, and other regulatory markers, which promotes the expansion of preexisting Tregs and the de novo generation of antigen-specific Tregs in an *Itgb8*-dependent manner. Meanwhile, EPOR^+^ cDC1s migrate to lymph nodes, where they limit the priming of T cells, thereby suppressing immunostimulatory responses. Conversely, conditional knockout of EPOR in cDC1s shifts their phenotype toward immunostimulatory maturation, characterized by increased expression of costimulatory molecules such as CD40 and decreased PD-L1. In the context of tumor immunity, EPOR-deficient cDC1s that have acquired tumor antigens can migrate to tumor-draining lymph nodes to induce precursor exhausted T cells, which subsequently promote the effector differentiation of tumor-infiltrating CD8^+^ T cells, thereby enhancing antitumor immunity. In this way, cDC1s centrally orchestrate the dynamic yin-yang equilibrium of antigen-specific tolerance versus activation (Illustration was created with BioRender.com). EPOR, erythropoietin receptor; cDC1, type 1 conventional dendritic cell; Treg, regulatory T cell; TLI/ATS, total lymphoid irradiation/antithymocyte serum; CCR7, C-C chemokine receptor type 7; PD-L1, programmed death-ligand 1; EPO, erythropoietin; TGF-β, transforming growth factor-β; *Itgb8*, integrin subunit beta 8.

While this study substantially advances our understanding of how cDC1s dictate immunostimulatory versus tolerogenic responses and identifies EPOR as a promising target for DC-centered therapies, key mechanistic and translational gaps remain unresolved. First, the signals that regulate EPOR expression in cDC1s are not fully defined. Although EPOR expression in cDC1s appears relatively modest under steady-state conditions, it can be dynamically up-regulated after TLI/ATS treatment. Accumulating evidence suggests that environmental cues can regulate DC function. For example, apoptotic cells have been shown to preferentially induce tolerance and repress genes associated with immunostimulatory maturation [7]. However, this study indicates that efferocytosis-induced tolerogenic maturation requires EPOR signaling. Thus, the precise regulatory interplay between apoptotic-cell clearance and EPOR expression needs to be further investigated. Beyond biochemical signals, physical cues in the microenvironment may also regulate the maturation state of cDC1s. For instance, tissue shape sensing can modulate CCR7 expression and tolerogenic potential in DCs, indicating that some mechanical factors may also influence EPOR expression [8]. Moreover, it will be necessary to examine whether specific mechanisms within the tumor microenvironment actively up-regulate EPOR on cDC1s to drive immune evasion. Because EPOR^+^ and EPOR^−^ states appear to represent reversible functional phenotypes rather than a stable population, elucidating the factors that control EPOR expression may enable targeted reprogramming of cDC1 function to improve DC-based immunotherapy. Mechanistically, the pathways by which EPOR signaling drives the tolerogenic maturation and function of cDC1s remain incompletely understood. Notably, EPOR up-regulation is accompanied by activation of mTOR signaling and lipid metabolic gene programs. Given that mTOR-dependent metabolic reprogramming has been linked to the functional properties of tolerogenic DCs, it is possible that EPOR signaling may promote tolerogenic programming through coordinated metabolic and transcriptional regulation [9]. At the level of cDC1–T cell interaction, integrin β8 has been identified as a critical downstream effector. However, the pathways linking EPOR activation to *Itgb8* expression, precursor-exhausted T cell formation, and suppression of effector T cell responses require further investigation. In addition, the original study shows that EPOR deletion in cDC1s reduces cell numbers slightly at steady state, but much more markedly following TLI/ATS treatment. This pattern suggests that EPOR may play a role not only in tolerogenic maturation but also potentially in cDC1 precursor maintenance or differentiation under inflammatory conditions. Future investigation into the function and mechanisms of EPOR in cDC1 precursor populations would therefore be informative.

From a translational perspective, these findings provide important guidance for cancer therapy. Erythropoiesis-stimulating agents have long been used to treat anemia in cancer patients, but their use has been clinically associated with increased tumor metastasis and mortality [10]. This study provides a mechanistic explanation for these observations by showing that EPO enhances EPOR signaling in cDC1s, thereby promoting antigen-specific immune tolerance. In parallel, the finding that loss of EPOR in cDC1s enhances the efficacy of immune checkpoint blockade supports the development of EPOR inhibitors as a potential therapeutic strategy. Conversely, EPOR signaling on cDC1s could be harnessed to induce immune tolerance in contexts, such as transplant rejection or autoimmune diseases. Several EPOR agonists have already been developed, and this work offers mechanistic insights to guide their rational application. Nevertheless, as the tolerogenic reprogramming described in the original study was established mainly in a TLI/ATS-based model, further studies are needed to determine whether the same mechanisms operate in other tolerogenic settings.

More broadly, the study highlights cDC1s as critical orchestrators of immune activation and effector function. This also underscores the limitations of strategies that aim solely to enhance cDC1 activity. Notably, despite up-regulation of CCR7 and enhanced migratory capacity, EPOR^+^ cDC1s preferentially induce antigen-specific Tregs in lymph nodes, leading to immune tolerance rather than immunity. Therefore, future immunotherapies should focus on directing DC functional states rather than solely enhancing antigen delivery or the migration of administered DCs.

## Data Availability

Not applicable.
